# Software for optimization of SNP and PCR-RFLP genotyping to discriminate many genomes with the fewest assays

**DOI:** 10.1186/1471-2164-6-73

**Published:** 2005-05-16

**Authors:** Shea N Gardner, Mark C Wagner

**Affiliations:** 1 Pathogen Bio-Informatics, Lawrence Livermore National Laboratory, Livermore, CA, USA

## Abstract

**Background:**

Microbial forensics is important in tracking the source of a pathogen, whether the disease is a naturally occurring outbreak or part of a criminal investigation.

**Results:**

A method and SPR Opt (**S**NP and **P**CR-**R**FLP **Opt**imization) software to perform a comprehensive, whole-genome analysis to forensically discriminate multiple sequences is presented. Tools for the optimization of forensic typing using Single Nucleotide Polymorphism (SNP) and PCR-Restriction Fragment Length Polymorphism (PCR-RFLP) analyses across multiple isolate sequences of a species are described. The PCR-RFLP analysis includes prediction and selection of optimal primers and restriction enzymes to enable maximum isolate discrimination based on sequence information. SPR Opt calculates all SNP or PCR-RFLP variations present in the sequences, groups them into haplotypes according to their co-segregation across those sequences, and performs combinatoric analyses to determine which sets of haplotypes provide maximal discrimination among all the input sequences. Those set combinations requiring that membership in the fewest haplotypes be queried (i.e. the fewest assays be performed) are found. These analyses highlight variable regions based on existing sequence data. These markers may be heterogeneous among unsequenced isolates as well, and thus may be useful for characterizing the relationships among unsequenced as well as sequenced isolates. The predictions are multi-locus. Analyses of mumps and SARS viruses are summarized. Phylogenetic trees created based on SNPs, PCR-RFLPs, and full genomes are compared for SARS virus, illustrating that purported phylogenies based only on SNP or PCR-RFLP variations do not match those based on multiple sequence alignment of the full genomes.

**Conclusion:**

This is the first software to optimize the selection of forensic markers to maximize information gained from the fewest assays, accepting whole or partial genome sequence data as input. As more sequence data becomes available for multiple strains and isolates of a species, automated, computational approaches such as those described here will be essential to make sense of large amounts of information, and to guide and optimize efforts in the laboratory. The software and source code for SPR Opt is publicly available and free for non-profit use at .

## Background

Microbial forensics and epidemiology is important in tracking the source of a pathogen, whether the disease is a naturally occurring outbreak or part of a criminal investigation. Polymorphisms among isolates or strains provide information as to the origin, phylogenetic relationships, or transmission patterns of those isolates [1]. The 2001 anthrax attacks highlight the importance of rapid forensic identification of the source of an agent used in a bioterrorism event. Sequencing HIV fragments indicated that a Florida dentist probably infected at least six of his patients with HIV [2]. A series of court cases in Scotland center on accusations that hospital staff are transmitting methicillin resistant staphylococcus aureus to patients [3]. The Centers for Disease Control and Prevention has also developed a network for molecular subtyping, or fingerprinting, of foodborne pathogens [4]. Because SNPs, insertion/deletion mutations, or sequence repeats may affect or be linked with phenotypic traits such virulence or antibiotic resistance, analysis of variance in polymorphic markers may also contribute to improvements in the diagnosis and treatment of infectious diseases[5].

Increasing availability of genomic sequence data makes it possible to predict regions of a genome that display variation among strains or isolates [1]. In the event of a suspected biothreat outbreak, the agent would be completely sequenced; however, full genome sequencing may require weeks or more. Ideally, there should be information immediately available about the hotspots of variation, the key sequence regions or assays that can discriminate among the possible sources of the agent, based on existing sequence data. This demands a full-genome analysis of available sequence data to predict discriminating markers among strains or isolates. Knowledge of and validated assays to query these variable regions for genotyping analyses could then be used to rapidly classify an unknown isolate in terms of its relationship to the already-characterized strains. These results could be available within hours, long before full sequence information becomes available. Once full-sequence information is generated, reliable, automated tools are required to find how this sequence differs or is similar to other strains. Recently, Budowle and colleagues stated that there is a "need for an infrastructure with analytical tools and knowledge bases to rapidly provide investigative leads..." [6].

The needs described above demand a full knowledge of all the SNPs and fragment length polymorphisms (e.g. detectable by PCR-RFLP analyses) that distinguish known isolates. SNPs and PCR-RFLP analyses have been used extensively in genotyping for forensic and epidemiological applications [7-10]. Although extensive experimental bench work or human examination of multiple sequence alignments can illuminate such variations, non-automated analysis is tedious and error-prone, especially for long sequences or when more than a few sequences are available. Existing software programs related to forensics focus on human crime forensics, paternity investigation, and so on, and do not enable full-genome prediction of marker regions or predict the combinations of variable regions that facilitate maximal isolate discrimination with a minimal number of assays [11].

To address this need, we have developed an automated forensic pipeline for SNP and PCR-RFLP optimization, called SPR Opt (**S**NP **P**CR-**R**FLP **Opt**imization). To our knowledge, this is the first comprehensive, automated, computational tool that performs the following four steps:

1) identifies all SNPs or PCR-RFLP variations in a set of input sequences that can be as long as whole microbial genomes,

2) groups the co-segregating markers into haplotypes,

3) performs combinatoric analysis on the haplotypes to generate multi-locus solutions to maximally discriminate each of the input sequences from the others,

4) uses simulated annealing to find the best solutions using the smallest total number of haplotypes (that is, the fewest total assays) to discriminate all the input genomes to the maximum degree possible.

Both SNP and PCR-RFLP solutions computed by the SPR Opt software include multiple loci when necessary for maximal sequence discrimination. In this paper, input sequences may also be referred to as genomes. However, the input need not be complete genomes; it can be gene sequences or other fragments from a number of isolates. Here a SNP is considered to be a single polymorphic base surrounded by conserved upstream and downstream sequence [9]. The length of the conserved sequence surrounding a SNP is specified by the user. In future versions of the software under development, a less strict SNP definition will be allowed in which requirements for conservation surrounding the SNP position are relaxed, allowing polymorphisms and indels in the region immediately surrounding the SNP position.

SPR Opt also predicts PCR-RFLP assays to discriminate the input sequences. Those primer pair and restriction enzyme combinations to maximally discriminate the input sequences are identified. For the PCR step, it is assumed that primer pairs must be conserved among all the input sequences. The resulting amplicons are examined for length polymorphisms or sequence variations that would result in differences in fragment length distributions after restriction digest among the multiple input sequences (RFLP). This software elucidates all PCR-RFLP detectable variations, whether they are caused by insertions or deletions of non-repeated sequence, tandem repeats, non-tandem repeats, microsatellites, mutations that alter a restriction site, and so on.

For both SNP and PCR-RFLP analyses, some conservation is required among the input sequences surrounding the variable site, whether it be conserved bases up and downstream of the SNP, or conserved primers surrounding the fragment length polymorphism or RFLP. This ensures that there are not false negatives because the surrounding sequence was not amplified or otherwise detected. For example, detection of SNPs may be performed using a microarray with oligos chosen so that the central base is a SNP, and there are 4 oligos for each conserved surrounding sequence, each representing the SNP position filled with A, T, C, or G, respectively. If there is not a hybridization signal from any of the four oligos, then this serves as an alert that there may be a problem with the reaction conditions or the sample. In contrast, if conserved oligos (sequences surrounding the SNPs) are not used, then it is unclear whether the sample is a variant without that particular SNP, or whether the sample is degraded, the reaction conditions unsuitable, or the target species is not present.

Here it is assumed that a haplotype is a group of markers (SNP or PCR-RFLP) that segregate in the same pattern across the genomes. Any single marker in a given haplotype indicates the identities of the other markers in this haplotype, so that every marker within the haplotype need not be examined. Here it is not assumed that the markers within a given haplotype cluster spatially in any pattern within a given sequence. That is, markers in the same haplotype may be distributed evenly or randomly, and do not necessarily occur within blocks of contiguous sequence. Haplotypes do not necessarily correlate with "hotspots" of mutation. Markers in the same haplotype may be likely to be linked, located in close proximity and inherited as a group, but it is not assumed that this is the case. Indeed, if markers in the same haplotype are separated in the genome, it may indicate that a recombination event has occurred. Alternatively, this pattern could be a result of selection or chance. Such an investigation of how positional information of markers in the same haplotype may indicate recombination is beyond the scope of this paper.

Although the first part of this software depends on locating the SNP or PCR-RFLP sites (step 1 above), the "backend" (steps 2–4 above) of this computational forensics pipeline depends only on the haplotypes. Thus, while the tools are currently coded to work for the SNP or PCR-RFLP analyses described here, any type of marker such as microarray hybridization patterns, microsatellite markers, or even chemical or physical features that differ among isolates could be categorized into haplotypes and fed into the back end of the combinatorial analyses described below.

One question that is investigated here is that of how well phylogenetic trees based on the haplotype splits of PCR-RFLPs or SNPs correspond with those generated from full genome sequence alignments or phenotypic differences. The results presented indicate that trees built solely on forensic markers do not closely match those based on full genome alignments. This finding has important implications for tree generation and interpretation in the absence of sufficient genomic sequence information, since empirical forensic techniques are often used for tree prediction. A second analysis presented is the frequency that different restriction enzymes result in fragment length polymorphisms for the SARS and mumps data.

The impacts of this software are the following: 1) The software provides computational guidance as to an optimal set of assays for genotyping the isolates, particularly helpful when large numbers of sequences or genomes are available. 2) All the SNP or PCR-RFLP variations in the available data are found and grouped into haplotypes. This includes identification of the sequence surrounding a SNP that will be useful in designing the assay (for example, the oligos or primers for an array, ligation, or single base extension reactions), or primer prediction and restriction enzyme selection. If the available sequence data is limited, there is no guarantee that the primers identified will be found in unsequenced strains, although requiring conservation among the sequenced isolates increases the chances of conservation among unsequenced isolates as well.

The application of SPR Opt is illustrated for two viruses for which multiple genomes are publicly available. The software and source code is publicly available and free for non-profit use at .

## Results

The output files listed in Table 2 containing the details of these analyses are provided as supplementary information for the web . The main results are summarized in Tables 3, 4, 5. The large number of genomes, variable sites, and character haplotypes illustrate the utility of this software for focusing in on the most informative combinations of sites. Only some of the mumps virus or SARS virus genomes can be uniquely discerned from other genomes of the same species. Unresolved clusters are given in Additional file 2. Computational prediction of PCR amplicon length variation (without restriction digest) for mumps virus indicates there are no variable length amplicons using the parameter values and definitions used here. Therefore, this method when restriction digest is omitted is not appropriate for genotyping mumps virus.

**Table 2:** Description of the output files created. Examples for the organisms analyzed here can be found at

**File Name**	**Content Description**
**SNPs_all, FLPs_all**	list of all the SNPs or PCR-RFLPs found in the input genomes
**genome_groups**	lists the genome groupings that correspond to each of the character haplotypes
**character_haplotypes**	lists the co-segregating SNPs or PCR-RFLPs that characterize each haplotype
**all_discriminating_sets**	for each genome, lists haplotype combinations ("sets") that will discriminate the specified genome to the maximum degree possible
**sim_anneal_results_summary**	lists combinations of sets of haplotypes to discriminate all the input genomes to the maximum degree possible using the fewest haplotypes. Each row is a unique combination that has the best score found, where the score is the number of haplotypes required.

**Table 3:** Summary of predictions for SNPs

Organism	Number Sequences	Number Sites	Number Haplotypes	Number Unresolved clusters	Minimum Number Assays
Mumps	17	171	85	11	10
SARS	102	218	164	65	114

The minimum number of assays indicates the fewest haplotypes that need to be queried to discriminate all the input genomes to the maximum degree possible, that is, the number of assays that would need to be done to classify an unknown isolate in terms of its similarity to the currently sequenced strains using this type of forensic marker.

**Table 4:** Summary of predictions for PCR without restriction digest

Organism	Number Variable Amplicons	No. Combinations of Amplicon X Unique Fragment Length Distributions	No. Haplo-types	No. Unresolved clusters	Min. No. Assays
Mumps	0	0	0	1	NA
SARS	39	178	51	26	31

The number combinations of Amplicon X Unique Fragment Length Distributions is the number of unique fragment length distributions summed over all variable amplicons.

**Table 5:** Summary of predictions for PCR-RFLP

Organism	Number Variable Amplicon X Enzyme Combinations	Number Combinations of Amplicon X Enzyme X Unique Fragment Length Distributions	No. Haplo-types	No. Unresolved clusters	Min. No. Assays
Mumps	1,070	3,113	288	13	13
SARS	1,694	7,344	440	75	99

The number of combinations of Amplicon X Enzyme X Unique Fragment Length Distributions is summed over all variable amplicon, enzyme combinations in the PCR-RFLP analyses.

**Figure 2:**
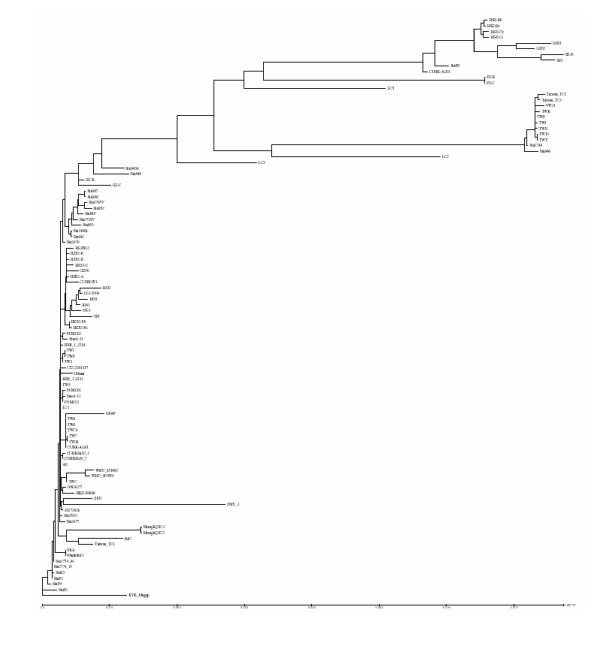
Phylogram of SARS created based on a multiple sequence alignment.

**Figure 3:**
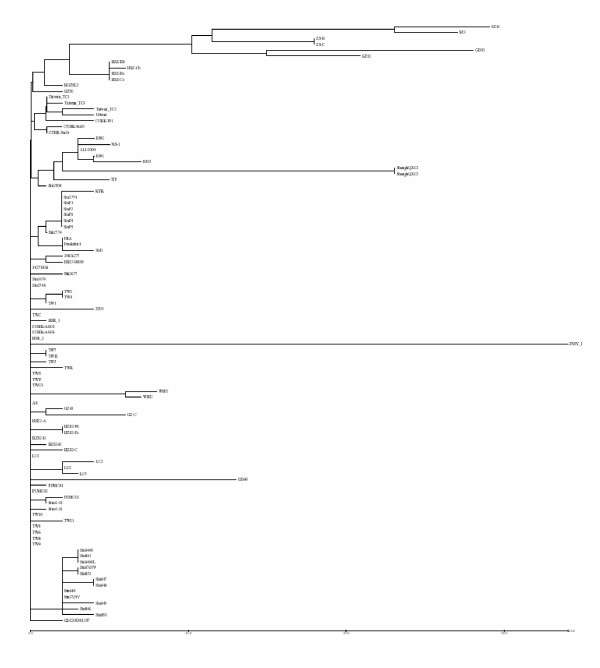
Phylogram of SARS created based on all SNP haplotypes.

**Figure 4:**
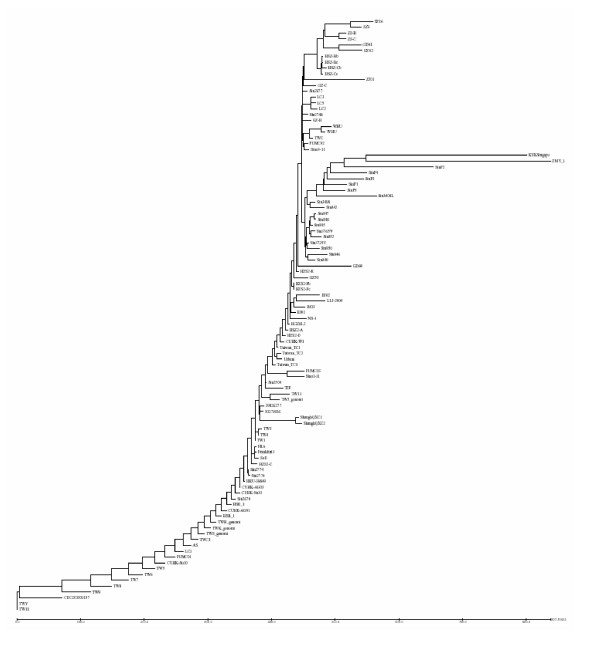
Phylogram of SARS created based on all PCR-RFLP haplotypes.

**Figure 5:**
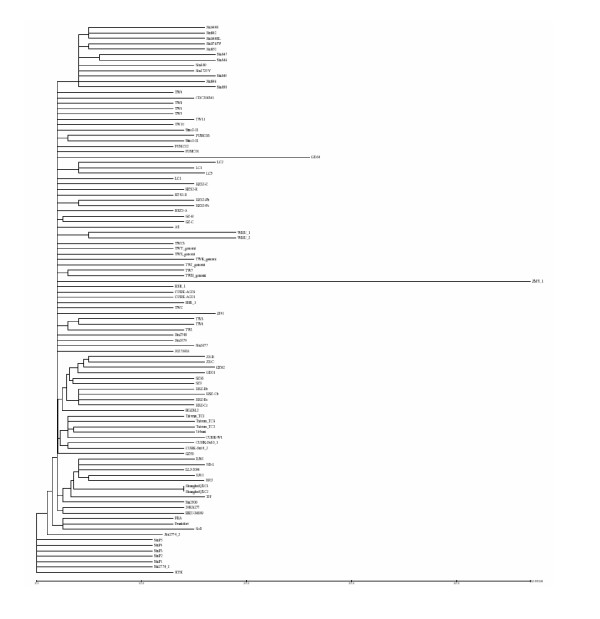
Phylogram of SARS created based on only SNP haplotypes in one optimal solution set (that maximally discriminates each of the input genomes using the fewest total haplotypes).

A comparison of the SARS phylograms created using full genome sequences, SNPs, or PCR-RFLPs (Figures 2, 3, 4, 5) illustrate that although there are similarities within the fine branch structure showing the relationship of very similar genomes, the basic structure of the trees differs. Genetic distances, indicated by the lengths of the branches, vary among the trees, and the particular genome clusters predicted to be the least similar (longest branches) contrast among the trees. A tree created from an optimal solution for SNP forensics has little branching structure and homogeneous branch lengths, and does not appear very similar to or to provide as much information as trees created from all the SNP data or from the multiple genome alignment.

Table 6 shows that the ranking of enzymes that most frequently generate RFLP variations in SARS virus are similar but not identical to the rankings for generating RFLP variations in mumps virus.

**Table 6:** Enzyme frequency for creating fragment length polymorphisms in SARS and mumps viruses

Enzyme	SARS	Mumps	Enzyme	SARS	Mumps
Tru9I	335	149	NciI	54	32
Hsp92II	320	98	EcoRV	52	36
Rsal	318	101	TthlllI	52	15
DdeI	317	104	EclHKI	52	11
Alul	270	135	Csp45I	51	29
Bsp1286I	227	48	Bbul	47	14
TaqI	213	121	SphI	47	14
CfoI	195	54	Bst98I	44	20
HhaI	195	54	XbaI	41	28
MboI	190	135	XmnI	39	29
Sau3AI	190	135	NheI	39	14
HinfI	179	105	AccB7I	39	8
AccI	170	45	NcoI	35	8
VspI	161	62	HpaI	34	8
BstOI	160	77	EcoRI	32	23
MspAI	140	55	AvaI	30	32
Styl	133	39	NruI	24	4
Sau96I	132	74	BclI	23	33
AvalI	128	57	HindIII	22	27
SinI	128	57	Sall	21	15
HincII	126	24	BssHII	20	4
BsrSI	119	49	Eco72I	19	6
RvuII	114	40	Smal	17	12
Dral	113	26	Xmal	17	12
Pstl	113	13	XhoI	13	14
XhoII	112	69	Mlul	13	0
Haell	112	18	Clal	9	12
HaelIl	110	83	SnaBI	8	4
Scal	103	16	Nael	6	8
HpaII	91	82	NgoMI	6	8
MspI	91	82	Eco47III	3	18
Sspl	91	33	Stul	3	17
BanII	91	18	BamHl	0	24
EcoICRI	91	8	AcyI	0	18
Spel	85	21	BalI	0	18
Ndel	83	20	Hsp92I	0	18
Nsil	82	8	Bgll	0	12
Acc65I	74	9	Agel	0	11
KpnI	74	9	BsaOI	0	9
BstXI	73	6	SacI	0	6
Bsu36I	72	16	AatII	0	4
BsrBRI	65	41	Apal	0	4
BglIl	64	35	Pvul	0	4
BanI	62	17	BstZI	0	3
BstEII	61	8	Eco52I	0	3
Alw441	59	6	SacII	0	2

## Discussion

PCR without restriction digestion was inferior to PCR-RFLP and SNPs for genome discrimination of the viruses examined, although in additional analyses of bacterial genomes, PCR without digestion was adequate for complete genome discrimination (unpublished). When it is possible to choose from a very large number of restriction enzymes, PCR-RFLP may enable a greater level of discrimination than SNP analysis, illustrated by the result that the genomes can be subdivided into more, smaller unresolved clusters using PCR-RFLP than using SNPs. However, this is not always the case: for example the two SARS genomes PUMC03 and Sino1-11 can be discriminated using SNPs but not using PCR-RFLP. If only a small number of restriction enzymes are available, then analyses using SPR Opt indicate that SNPs outperform PCR-RFLP (unpublished). In most cases, either SNPs or PCR-RFLPs can discriminate most genomes, with minor differences in the exact unresolved clusters predicted. In these cases, one may need to use both techniques in order to discern different sets of similar genomes.

Contrasting the phylogenetic trees generated from full genomes, SNPs, or PCR-RFLPs suggests that caution is needed in assessing genome divergence based on forensic/epidemiological data in lieu of full genome sequences, since the isolates predicted to be most divergent may differ across the three measures of variation. In addition, if only the subset of SNPs determined to be optimal for forensic discrimination are queried, phylogenetic trees based on this information may not be representative of the true phylogenic relationships, and may result in particularly poor estimation of branching structure and branch lengths.

Highly heterogeneous viruses like many of the single-stranded RNA viruses (e.g. human immunodeficiency virus, hepatitis virus species, poliovirus, etc.) must be subdivided into clades or other sub-groupings of genetically similar strains even before this software can be applied. Lack of conserved sequence upstream and downstream of a SNP, or lack of conserved primers for PCR amplification, may thwart attempts at SNP or PCR-RFLP discovery at the species level. Instead, one requires subgroups of genomes that are sufficiently similar to locate conserved regions surrounding the forensically informative sites. A future version of the software under development will employ a less restrictive definition of SNPs, allowing some variation surrounding the SNP position.

In addition to SNP and PCR-RFLP analysis like the examples presented here for a given target species, there are many questions one could address with the aid of this software. For example, the character haplotypes and genome groups generated by SPR Opt contain a wealth of information. If there are differences in virulence, host range, or other interesting phenotypic traits, one may examine the list of genome groups to see if there are any whose genome membership corresponds with the phenotypic variation. If so, these may be interesting regions for further biological investigation. For example, in which genes do the corresponding character haplotypes land? Do the nucleotide variations translate into protein sequence differences? One could also take a complementary approach, asking whether SNPs or PCR-RFLPs are clustered in certain genes or intergenic regions. It would also be interesting to examine whether or not SNP locations within a species correspond to regions of relative inter-specific conservation or variation. As mentioned in the introduction, the distribution of co-segregating markers in a given haplotype across the genome sequence might be used to look for evidence of recombination events or correlated selection on multiple genes, as might be observed if genes are in the same pathway or affected by the same environmental factors.

## Conclusion

In conclusion, bioinformatic software called SPR Opt is described to optimize SNP and PCR-RFLP analyses in order to provide the maximum amount of genotyping information from the fewest assays at the bench. SPR Opt requires as input a set of sequences and their multiple sequence alignment. This software not only predicts the variable sites based on input sequence data, but also groups these into co-segregating haplotypes and provides guidance as to the ways in which these may maximally discriminate genomes using the fewest possible assays. These are computationally challenging problems that are solved using a bit vector intersection approach to determine sets of haplotypes to maximally discriminate each input genome, as well as parallel simulated annealing to select a subset of the many possible solutions that will enable users to extract the most information from the fewest forensic tests. Analyses of two viruses were presented, and a number of potential investigations using this software are suggested. This is the first comprehensive tool to optimize the selection of forensic markers to maximize information gained from the fewest assays, accepting whole or partial genome sequence data as input. As more sequence data becomes available for multiple strains and isolates of a species, automated, computational approaches such as those described here will be essential to make sense of large amounts of information, and to guide and optimize efforts in the laboratory.

## Methods

### The input required

The required input to the software is a fasta-formatted file of all the input genomes and a multiple sequence alignment. A consensus "gestalt" is automatically constructed from the multiple sequence alignment of the input genomes, in which conserved bases are indicated as letters (A, C, G, or T) and dots indicate positions where all input sequences do not agree [12]. Where there are insertions or deletions in the alignment, the corresponding positions in the conservation gestalt contain dots.

#### SNPs

A SNP is defined to be a single polymorphic base surrounded by conserved upstream sequence of length *min_len_upstream *bases and conserved downstream sequence of length *min_len_downstream *bases. By conserved sequence, it is meant that the sequence is identical among all input genomes. If the sequence surrounding a SNP occurs more than once or is absent in any of the input genomes (checking both the plus and minus strands), the position is deleted from consideration as a SNP. A polymorphic base differs in one or more of the input genomes. If *min_len_downstream *and *min_len_upstream *are greater than zero, then this is a strict definition of SNPs. Insertions or deletions of even a single base are not considered SNPs. The parameters *min_len_upstream *and *min_len_downstream *are user-specified. The software finds SNPs using the conservation gestalt; in the conservation gestalt, any dot surrounded by at least *min_len_upstream *and *min_len_downstream *letters is a candidate SNP. The sequences indicated by the letters upstream and downstream of the candidate SNP are stored. Using regular expression pattern matching in PERL, the location of the SNP is determined in each of the input genomes. If an exact match to the conserved sequence surrounding the SNP does not occur exactly once in each of the input sequences, then the SNP and its surrounding sequence is eliminated from further consideration. Thus, the SNP is defined by its conserved surrounding sequence, and the position of a SNP within a genome may differ among the genomes.

If a less restrictive definition of a SNP is required, it is possible to set either (but not both) *min_len_downstream *or *min_len_upstream *equal to 0, and thus pick up more regions, including those that may be the beginning of insertion/deletion variations among genotypes. This might be a desired approach for the Single Base Extension assay, since only conservation immediately 5' of the variable position matters [9].

The choice of the length requirement for the surrounding conserved sequence is very important. If *min_len_upstream *and *min_len_downstream *are too short, then sequence surrounding the SNP is likely to be repeated within a given genome. Since each of the repeats could have a different character at the SNP position, if these candidates are not eliminated then it is not possible to distinguish the case of a sample containing a mixture of multiple genotypes from the case of a sample containing a single strain that has multiple occurrences of the sequence surrounding a candidate SNP position with different bases filling the variable position. In contrast, if *min_len_upstream *and *min_len_downstream *are too long, then SNP variation may be overlooked because candidate SNPs are surrounded by an insufficient number of conserved bases. If a microarray chip platform using oligos of length 25 bases is to be used for the assay, then setting *min_len_upstream *and *min_len_downstream *equal to 12 will predict 25-mers with the central base position the SNP.

This software excludes degenerate bases (e.g. R = G or A, Y = T or C, etc.) indicated within a given genome from consideration as SNPs. Degenerate bases may be due to polymorphic populations within a given strain or to low quality sequencing, and thus do not deliver a confident characterization of differences between strains.

#### PCR-RFLPs

A PCR-RFLP variation occurs if PCR amplicons or the fragments that result after restriction digest of such amplicons have a different length distribution among the input genomes. Fragment length distributions differ if they have different numbers of fragments or if any of the fragments differ in length.

The fragments are generated by first determining amplicons in which the forward and reverse primers are conserved among all the input genomes. If no restriction digest is to be applied, then an amplicon must differ in length among the input genomes. With restriction digest, the distribution of fragment lengths after cutting the amplicon with restriction enzymes must differ among the genomes. The parameter *num_restriction_enzymes *may be set equal to 0, 1, 2, or 3, as will be described below. Length variation must be detectable by electrophoresis, since there is a limit to the precision to which lengths can be determined. Here, if all the fragments in each of the genomes are less than 50 bases long, or if there are no differences in fragment lengths among any of the genomes that are at least *precision *bases, then the amplicon+enzyme combination is not considered to be sufficiently variable.

In order to find candidate regions for PCR-RFLP variation, sequence fragments that may contain a PCR-RFLP marker are selected from the conservation gestalt. These are chosen to be just over the *max_amplicon_length*, to have at least one variable position (a dot in the gestalt file), and are chosen using a sliding window moving at least *jump *bases from the start of the previous window. If *jump *is too large, PCR-RFLP sites may be missed, and if *jump *is too small, the same insertion/deletion variation may be counted more than once if it is contained within more than one pair of primers. However, all instances of counting the same variable site will appear within the same haploblock, so a simple examination of the markers within a given haploblock sorted by position should indicate those that query the same site as a result of using too small a value for the *jump *parameter. The software may also be run several times with different values of *jump*, and the largest value of *jump *that still provides the greatest discrimination among genomes may be selected.

Second, conserved primer pairs are selected from these fragments using MIT's primer3 software with user-specified parameters. Third, genomes are searched for an exact match to each primer pair on both the plus and minus (reverse complemented) strands, and those primer pairs are discarded in which 1) one or both primers are absent from any of the genomes, 2) one or both primers occur more than once in any of the genomes (with no mismatches), or 3) the forward and reverse primers are too far apart to reliably generate an amplicon in one or more of the genomes. In the analyses here, it is assumed that any distance longer than 1200 bases is too long for amplification, which is reasonable if a short elongation time in the PCR thermocycle is to be used. This value can be changed in the source code.

Amplicons can be cut by 0, 1, 2 or 3 enzymes simultaneously (or sequentially before being run through electrophoresis), as a user-specified option *num_restriction_enzymes*. Simulated cutting is performed computationally using the regular expression pattern matching function in Perl, and it is assumed that cuts occur in all locations where a given enzyme sequence occurs (that is, the DNA is exposed to the enzyme for a sufficient duration to cut at all the sequence-specific sites).

If the number of restriction enzymes is set to 0, then in the output files the restriction enzyme is indicated as "NONE", and the PCR amplicons must vary in length among the sequences without any restriction digest. If *num_restriction_enzymes *= 1, then a given PCR product may be digested by only one enzyme at a time before electrophoresis or other empirical measurement of fragment lengths. That is, digestion of the original PCR products with many alternative enzymes may be performed as long as each digestion is followed by its own fragment length measurement. The final solution guiding how to discriminate all the input sequences may involve a number of different enzymes. But when *num_restriction_enzymes *= 1, digestion is always done only with one enzyme at a time before fragment lengths are measured.

If *num_restriction_enzymes *= 2 or 3, then digestion can occur with combinations of 2 or 3 enzymes, respectively, before the measurement of fragment lengths occurs after each digestion with a given combination of enzymes. Enzyme combinations should be examined by the user to make sure the buffers and reaction conditions are compatible for all the enzymes in the combination, as the software does not assess enzyme compatibility. Although setting *num_restriction_enzymes *= 2 or 3 is allowed, it is not clear that users ever need to use these options, since with *num_restriction_enzymes *= 1, many different enzymes may contribute to the total solution of discriminating all the input genomes, as long as they are not applied simultaneously. All results reported here were computed using *num_restriction_enzymes *= 1.

The restriction enzymes to be considered are specified by the user. Currently, the software is not implemented for non-palindromic restriction enzymes, although this could be added as a minor modification if required.

### Procedures implemented by the software

#### 1) Find all SNP or PCR-RFLP sites

First, the software calculates all SNPs or all PCR-RFLPs, as defined above. These are listed in the files *SNPs_all *or *FLPs_all*, respectively. "FLP" refers to Fragment Length Polymorphisms, whether the polymorphisms result from PCR amplification or restriction digest length differences. The total number of SNPs is counted as the number of positions that are variable. For PCR-RFLPs, the software reports the total number of unique combinations of primer pair sequences and restriction enzyme(s) that yield variation in fragment length distributions among the genomes, as well as the number of unique fragment length distributions (summed across primer pairs and restriction enzyme combinations).

#### 2) Cluster SNPs or PCR-RFLPs into co-segregating groups called haplotypes

The second computation the software performs is to divide the SNPs (or PCR-RFLPs) into co-segregating groups of markers called character haplotypes. That is, any two variable markers that distinguish the same set of genomes, and thus provide equivalent forensic/epidemiological information, are considered members of the same character haplotype. The genomes that co-segregate for a particular character haplotype is called the genome group. Genomes are members of multiple genome groups that may overlap in membership, and there may be genome groups that are proper subsets of other genome groups. For easy association, the genome group identification number is the same as the character haplotype identification number. The algorithm to generate the list of character haplotypes and genome groups works in the following way: at each variable site, genomes are grouped by the marker identity ("allele", e.g. SNP character) each genome contains at that locus. Every time a new clustering pattern of genomes occurs, the cluster of genomes defines a new genome group, and the marker is stored in the character haplotype associated with that genome group. If a clustering pattern has already been observed at a locus previously examined, then that allele is added to the list of markers for the associated character haplotype.

The file *character_haplotypes *contains a listing of the SNPs or PCR-RFLPs contained in each character haplotype, and the file *genome_groups *gives the associated genome groups. Inspection of these files is useful in additional analyses of sequence clusters (e.g. phylogenetic tree construction based on number of SNPs or PCR-RFLPs supporting a given relationship) or group-level assays (find a haplotype that will distinguish any of genomes A, B, or C from all the others, e.g. discriminate virulent strains from vaccine strains).

#### 3) Find sets of haplotypes that maximally discriminate each genome or unresolved cluster of genomes

The third part of the software computes all sets of 1 or more character haplotype(s) that will maximally discriminate each genome. Each genome requires the testing of one or more polymorphic sites to pull it out from the other genomes. Thus, each solution set to resolve a given genome contains one to many haplotypes (a multi-locus solution). There may be many alternative solution sets for every genome, each of which provide the same information, and it is up to the user to select the one that works the best on the chosen platform.

To find a set of character haplotypes to uniquely discriminate one genome from the rest requires that the intersection of the genomes across the associated genome groups is that single target genome. For example, if genome group 1 (associated with haplotype 1) contains genomes A, B, and C, and genome group 2 (associated with haplotype 2) contains genomes A and D, then the set of haplotypes 1 and 2 can uniquely discriminate genome A. That is, if in an unknown sample, one found a SNP that was included in haplotype 1 and another SNP that was included in haplotype 2, then it would be concluded that the unknown sample was like A, and not B, C, or D.

The combinatoric demands of this step may be substantial. For example, if there are 100 genome groups, then examining the intersections of all possible combinations of 3 genome groups requires _100_C_3 _= 161,700 tests. We represent each genome group as a bit vector of 1's and 0's indicating the membership or exclusion, respectively, of each of the genomes in that genome group. Then all genome groups containing only a single genome (each of the markers in the associated character haplotype uniquely identifies that genome) are reported, as well as all genome group combinations of size = *number_combinations_to_test *and fewer in which the bitwise AND (intersection) is a single genome. The bit vector approach was the fastest method that was tested by the authors. Thus, all intersections of sets of genome groups are examined, and for each of the input genomes, a search is made for those in which the intersection is uniquely that genome. The maximum number of genome groups per combination that is tested in a multi-locus solution set is the parameter *number_combinations_to_test*. Thus, combinatorics are performed on _number haplotypess_C_number_combinations_to_test _total combinations.

If there is not an intersection that contains uniquely the target genome, the intersections that contain the fewest other genomes in addition to the target genome is reported as the solution set for that target. Thus, if the target in question cannot be uniquely discriminated, the most specific level to which it can be discerned is output. The other genomes in solution set *i *for genome A is represented as the list *others(i,A)*.

When there are many genomes and many genome groups, it may be necessary to take two additional steps to find the combinations to maximally discriminate sequences. For example, there may be hundreds of genome groups, so that the *number_combinations_to_test *must be set at the low value of 2 in order for the combinatoric step to finish in a reasonable amount of time (hours or less). First, if there are two preliminary solutions *i *≠ *j *where *others(i,A) *≠ *others(j,A)*, new combinatorics are performed on only the union of the genome groups that comprise all preliminary solutions for the genome A under consideration. Since the number of genome groups making up all preliminary solutions for the specific genome A is a small subset of the total number of genome groups, testing all possible combinations (not just combinations up to a given size) computes rapidly. This generates new solution sets, each of which contains more genome groups than the preliminary solution sets. Each of these new solution sets for the given genome has an identical list of *others(A)*, so that the index *i *may be dropped.

A second step is sometimes required to find maximally discriminating multi-locus combinations in situations with large numbers of genomes and genome groups. For genome B that is in the list of *others(A)*, if the size of *others(B) *< size of *others(A)*, then one should be able to discriminate genome A to a higher level (with fewer *others(A)*), if more genome groups are included in the solution set. In this case, the program cycles through all genome groups, adding the first genome group encountered to the solution sets for A that contain genome A but not genome B. Although taking the first acceptable genome group may not be the best (that is, may not cause the greatest reduction possible in the list *others(A)*), this method is fast and does give a good solution in the many test cases examined. The list of *others(A) *is then recomputed with the new genome group included in the solution set, and this process is repeated for all genomes in the list *others(A)*. We repeat this procedure for all the genomes that cannot be uniquely discriminated. This generates final solution set(s) for each genome that may contain many more genome groups than the original combinatoric size *number_combinations_to_test*.

The first step described above is performed automatically based on results of comparing lists of *others(i,A) *with *others(j,A) *for all *i *≠ *j*. The second step is computed automatically based on results of comparing *others(A) *with *others(B) *for all genomes A and B. These extra steps are rarely needed, since in most cases, there are few enough genome groups to select a sufficiently large value of *number_combinations_to_test *to find optimal solution sets with the fewest possible genome groups for each genome, rather than to find acceptable (but possibly sub-optimal) solution sets containing more than *number_combinations_to_test *genome groups. These steps were required for analyses of SARS virus. All solution sets for each genome are given in the file *all_discriminating_sets*.

There is a third option for speeding up the combinatorics of the process of finding sets of genome groups that maximally discriminate each genome: this is to exclude consideration of those genome groups with many genomes from the combinatoric calculations. This step yields a set of "pared groups". Since genome groups with many genomes provide the poorest discrimination among those genomes, it is reasonable to only consider the genome groups with the fewest genomes. This is an optional parameter *pare*_*groups *that can be set to 1. If *pare*_*groups *= 1 then the algorithm finds the cutoff number of genomes per genome group below which there are genome groups that together contain all the input genomes. Then only the haplotypes that contain fewer than this cutoff number of genomes are considered in the combinatoric steps described above. This option may result in orders of magnitude improvement in algorithm speed and memory for situations in which there are a large number of genome groups.

When genomes cannot be uniquely distinguished at the single genome level, the maximally discriminating genome groups are referred to as unresolved clusters. For complete isolate-level discrimination of all the input genomes, the number of unresolved clusters must equal the number of genomes.

#### 4) Find combinations of solution sets containing the fewest total haplotypes

**Figure 1:**
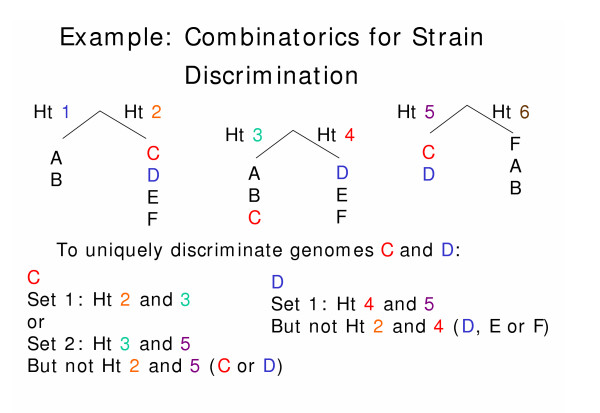
Combinations, or sets, of haplotypes can uniquely distinguish an individual genome if the intersection of the genomes across that set is uniquely the one genome in question.

Finally, the combinations of solution sets for each genome are found that enable the testing of the fewest total character haplotypes (i.e. that are associated with the fewest genome groups). For the example shown in Figure 1, either set 1 or set 2 can differentiate genome C. If set 1 is used, then membership in four haplotypes (Ht 2, 3, 4, and 5) must be queried to discern both C and D, but if set 2 is chosen instead, then only 3 haplotypes (Ht 3, 4, and 5) need to be examined. A similar analysis would take place to determine the minimum number of character haplotypes to maximally discriminate all the genomes A-F shown in Figure 1.

When many of the genomes can be discriminated by multiple alternative solution sets, the number of possible set combinations across all the genomes is the product of the number of solution sets for each genome. Unpublished analyses show that the number of possible set combinations can skyrocket to over 10^30 ^combinations, far too many to do an exhaustive search for the global optimum solution that minimizes the number of character haplotypes to be examined. Therefore, simulated annealing was used to search for approximately optimal solutions using the Metropolis algorithm. Forty parallel processes of simulated annealing were run, and from these the best solutions were selected. The best score is the minimum number of haplotypes that must be examined. The file *sim_anneal_results_summary *lists the combinations of set numbers (each set is a set of haplotypes) for each genome that enable the fewest haplotypes to be tested overall. Each row is a different solution from simulated annealing that has the same score, that is, each row contains a list of set numbers, one set for each genome, characterizing the combination of sets. The set numbers correspond to the set numbers given in the file *all_discriminating_sets*, and are not the same as the genome group or character haplotype numbers. Many of these simulated annealing solutions are very similar to one another. For test cases examined in which there were fewer than 10^7 ^possible set combinations, we verified that simulated annealing predicted the true global optimum.

### Parameters for analyses described here

For the analyses described below, the following parameter values were used for SNP analyses: *min_len_upstream *= 7 and *min_len_downstream *= 7. The minimum length of conserved upstream and downstream bases was selected to be 7 because it enabled a finer level of genome discrimination than did 12-mers (which would correspond with Affymetrix chip 25-mers with the central base being either a perfect match or a mismatch).

For the PCR-RFLP analyses described here, the *max_amplicon_length *= 1000. Amplicons of length 900–1000 bases are specified as preferred, although shorter amplicons are allowed if longer ones cannot be found. Other parameters are: *jump *= 500 when *num_restriction_enzymes *= 1, *jump *= 200 when *num_restriction_enzymes *= 0, and *precision *= 5. Primer3 parameters in the file p3.params.pcr.primers as well as a file listing the restriction enzymes used in the computations are available for download at .

SNP and PCR-RFLP analyses were performed using SPR Opt on 102 genomes of severe acute respiratory syndrome (SARS) virus (~30 Kb) and 17 genomes of mumps virus (~15 Kb) that were publicly available at the time the analyses were done. The Genbank genome identification information for these is given in Additional file 1. The multiple sequence alignments were generated using Multiple Genome Aligner [13].

### Phylogenetic analyses

Three phylogenetic trees for SARS virus were constructed based on 1) a full genome multiple sequence alignment, 2) SNPs, and 3) PCR-RFLPs. Newick trees were created using the PHYLIP software package [14,15], using the neighbor program to generate the trees based on a distance matrix of pairwise distances between the genomes. For the multiple sequence alignment, the dnadist program using maximum likelihood was used to generate the distance matrix. For SNPs and PCR-RFLPs, distance matrices were created by summarizing the data contained in the genome groups and character haplotypes. This was required because the raw listing of SNPs and PCR-RFLPs necessarily differed in format, while the genome group formatting was consistent between the SNP and the PCR-RFLP data so the same algorithm could be used to generate phylogenetic trees from each method. For the SNP data, the algorithm described below was validated by comparing the resulting phylogenetic tree with that created from a standard SNP matrix, also described below.

To calculate a distance matrix from the genome group data, first, each genome group was weighted by the number of SNPs or the number of PCR primers (for PCR-RFLP) contained in the associated character haplotype. For PCR-RFLPs, the weight was not increased if two or more alternative enzymes cutting the same amplicon (i.e. from the same set of primers) gave the same pattern of genome segregation, since in the runs described, there were many possible enzymes that provided the same information about genome relationships after cutting a single amplicon, and this might artificially increase the weight. After the weights were calculated, then for all possible pairs of genomes, each genome group was examined, and each time a genome group contained both of the genomes, the weight of that genome group was subtracted from the total distance score between that pair of genomes. After all the pairwise distances were calculated, a constant equal to the minimum (negative) distance was subtracted from each pairwise distance, so that none of the distances would be negative. The distance matrices for each of the SNP and the PCR-RFLP analyses were used in the PHYLIP neighbor program. The resulting phylogenetic trees (phylograms) were drawn using a web interface [16]. A phylogenetic tree was also created in a similar manner to that described above except based on only the SNP genome groups in one randomly chosen optimal set solution listed in the results from simulated annealing (that is, a set of the minimum number of haplotypes to maximally discriminate all the input genomes). Such a tree would represent a purported phylogeny if only the data from a set chosen for maximum forensic discrimination is used to predict phylogenetic relationships.

To verify that this method of creating a distance matrix from the genome group data provides an accurate picture for the SNP data, a distance matrix was created from a traditional SNP matrix. In the SNP matrix, columns correspond to the SNPs, and rows correspond to the isolates. This matrix, containing every SNP in the data set, was used in the dnadist algorithm of PHYLIP using the maximum likelihood option. The tree generated was identical to that created using the algorithm described above calculating distances from the genome groups.

### Restriction enzyme frequencies for creating RFLPs

The total number of times that each restriction enzyme created a different distribution of fragment lengths was summed across all amplicons in the PCR-RFLP analyses. This was done simply by counting the frequency of occurrence of each enzyme in the file *FLPs_all *(Table 6).

## Abbreviations

SPR Opt: SNP and PCR-RFLP Optimization

SNP: Single Nucleotide Polymorphism

PCR-RFLP: Polymerase Chain Reaction-Restriction Fragment Length Polymorphism

## Authors' contributions

SNG conceived of the project, developed much of the software, and drafted the manuscript. MCW created the code for performing set combinatorics using bit vectors, assisted with the code for distributed simulated annealing, and bundled the code for distribution.

**Table 1:** Description of the user-specified parameters for SNP and PCR-RFLP analyses

**User Specified Parameters**	**Description**
**min_len_upstream**	minimum length of conserved sequence upstream (5') of a SNP
**min_len_downstream**	minimum length of conserved sequence downstream (3') of a SNP
**max_amplicon_length**	maximum amplicon length allowed for PCR-RFLP analysis
**jump**	the series of sequences input to primer3 for amplicon generation for PCR-RFLP analyses are chosen by sliding a window along the consensus gestalt. Each new window must start at least jump bases from the start of the previous window.
**precision**	there must be at least one difference in fragment lengths among all input genomes that is at least this long for the given amplicon+enzyme combination to be considered variable enough for further PCR-RFLP examination.
**num_restriction_enzymes**	number of restriction enzymes used in an PCR-RFLP analysis before a single electrophoretic determination of fragment length distributions is made. This number may range from 0–3.
**number_combinations_to_test**	maximum number of haplotypes per combination tested in a multi-locus solution set to maximally discriminate all the input sequences. Thus, combinatorics are performed on _number haplotypess_C_number_combinations_to_test _total combinations.

## Supplementary Material

Additional File 2 Lists of unresolved clusters. **Description of additional data files provided at **Digest*organism_name NoDigest*organism_name SNP*organism_name The * refers to the files indicated in Table 2. Digest* is for PCR-RFLP with num_restriction_enzymes = 1, and NoDigest* is for PCR-RFLP with num_restriction_enzymes = 0. The NoDigest* results are not given for mumps, since there was not adequate variation using this method for forensic discrimination of these input sequences, as indicated in Table 4. The multiple sequence alignment files used in these analyses for SARS and mumps viruses are also available for download. There are a total of 27 files containing all the microbial forensic results and data described above. All are in text format, and can be found at . Click here for file

Additional File 1 List of genomes used in the analyses. **Description of additional data files provided at **Digest*organism_name NoDigest*organism_name SNP*organism_name The * refers to the files indicated in Table 2. Digest* is for PCR-RFLP with num_restriction_enzymes = 1, and NoDigest* is for PCR-RFLP with num_restriction_enzymes = 0. The NoDigest* results are not given for mumps, since there was not adequate variation using this method for forensic discrimination of these input sequences, as indicated in Table 4. The multiple sequence alignment files used in these analyses for SARS and mumps viruses are also available for download. There are a total of 27 files containing all the microbial forensic results and data described above. All are in text format, and can be found at . Click here for file
