# Supplementary material for: Software for optimization of SNP and PCR-RFLP genotyping to discriminate many genomes with the fewest assays
Source: BMC Genomics. 2005 May 16;6:73. doi: 10.1186/1471-2164-6-73 (PMC1156889; doi:10.1186/1471-2164-6-73)
Supplement: Additional File 2 — Lists of unresolved clusters. Description of additional data files provided at Digest*organism_name NoDigest*organism_name SNP*organism_name The * refers to the files indicated in Table 2. Digest* is for PCR-RFLP with num_restriction_enzymes = 1, and NoDigest* is for PCR-RFLP with num_restriction_enzymes = 0. The NoDigest* results are not given for mumps, since there was not adequate variation using this method for forensic discrimination of these input sequences, as indicated in Table 4. The multiple sequence alignment files used in these analyses for SARS and mumps viruses are also available for download. There are a total of 27 files containing all the microbial forensic results and data described above. All are in text format, and can be found at . [file 1471-2164-6-73-S2.doc]

Additional File 2: Lists of unresolved clusters

The following lists the unresolved clusters for SARS for SNP analyses, PCR analyses with no restriction digestion, and PCR-RFLP analyses.

SARS SNP analysis with min_len_upstream= min_len_downstream=7:

Number of unresolved clusters: 65

Unresolved cluster 1:

gi|37624342|gb|AY394999.1| SARS coronavirus LC2, complete genome

Unresolved cluster 2:

gi|33411429|dbj|AP006559.1| SARS coronavirus TWK genomic RNA, complete genome

Unresolved cluster 3:

gi|45645019|gb|AY559093.1| SARS coronavirus Sin845, complete genome

Unresolved cluster 4:

gi|38231927|gb|AY350750.1| SARS coronavirus PUMC01, complete genome

Unresolved cluster 5:

gi|31416292|gb|AY278487.3| SARS coronavirus BJ02, complete genome

Unresolved cluster 6:

gi|34482139|gb|AY304488.1| SARS coronavirus SZ16, complete genome

Unresolved cluster 7:

gi|37624321|gb|AY394978.1| SARS coronavirus GZ-B, complete genome

Unresolved cluster 8:

gi|37960831|gb|AY313906.1| SARS coronavirus GD69, complete genome

Unresolved cluster 9:

gi|30698326|gb|AY291451.1| SARS coronavirus TW1, complete genome

Unresolved cluster 10:

gi|38231937|gb|AY357076.1| SARS coronavirus PUMC03, complete genome

Unresolved cluster 11:

gi|35396382|gb|AY394850.1| SARS coronavirus WHU, complete genome

Unresolved cluster 12:

gi|33411399|dbj|AP006557.1| SARS coronavirus TWH genomic RNA, complete genome

gi|40548957|gb|AY502930.1| SARS coronavirus TW7, complete genome

Unresolved cluster 13:

gi|30027617|gb|AY278741.1| SARS coronavirus Urbani, complete genome

Unresolved cluster 14:

gi|37624333|gb|AY394990.1| SARS coronavirus HZS2-E, complete genome

Unresolved cluster 15:

gi|37361915|gb|AY283798.2| SARS coronavirus Sin2774, complete genome

gi|45645007|gb|AY559088.1| SARS coronavirus SinP1, complete genome

gi|45645010|gb|AY559089.1| SARS coronavirus SinP2, complete genome

gi|45645013|gb|AY559090.1| SARS coronavirus SinP3, complete genome

gi|45645016|gb|AY559091.1| SARS coronavirus SinP4, complete genome

gi|45645017|gb|AY559092.1| SARS coronavirus SinP5, complete genome

Unresolved cluster 16:

gi|49176846|gb|AY595412.1| SARS coronavirus LLJ-2004, complete genome

Unresolved cluster 17:

gi|37624335|gb|AY394992.1| SARS coronavirus HZS2-C, complete genome

Unresolved cluster 18:

gi|38505482|gb|AY485277.1| SARS coronavirus Sino1-11, complete genome

Unresolved cluster 19:

gi|31581502|gb|AY291315.1| SARS coronavirus Frankfurt 1, complete genome

gi|33578015|gb|AY310120.1| SARS coronavirus FRA, complete genome

Unresolved cluster 20:

gi|30271926|ref|NC_004718.3| SARS coronavirus, complete genome

gi|30468044|gb|AY283796.1| SARS coronavirus Sin2679, complete genome

gi|30468045|gb|AY283797.1| SARS coronavirus Sin2748, complete genome

gi|31873092|gb|AY321118.1| SARS coronavirus TWC, complete genome

gi|33114190|gb|AY345986.1| SARS coronavirus CUHK-AG01, complete genome

gi|33114214|gb|AY345988.1| SARS coronavirus CUHK-AG03, complete genome

gi|33115118|gb|AY323977.2| SARS coronavirus HSR 1, complete genome

gi|33411444|dbj|AP006560.1| SARS coronavirus TWS genomic RNA, complete genome

gi|33411459|dbj|AP006561.1| SARS coronavirus TWY genomic RNA, complete genome

gi|33518725|gb|AY362699.1| SARS coronavirus TWC3, complete genome

gi|37576845|gb|AY427439.1| SARS coronavirus AS, complete genome

gi|37624326|gb|AY394983.1| SARS coronavirus HSZ2-A, complete genome

gi|37624332|gb|AY394989.1| SARS coronavirus HZS2-D, complete genome

gi|37624341|gb|AY394998.1| SARS coronavirus LC1, complete genome

gi|38231932|gb|AY357075.1| SARS coronavirus PUMC02, complete genome

gi|40548873|gb|AY502923.1| SARS coronavirus TW10, complete genome

gi|40548933|gb|AY502928.1| SARS coronavirus TW5, complete genome

gi|40548945|gb|AY502929.1| SARS coronavirus TW6, complete genome

gi|40548969|gb|AY502931.1| SARS coronavirus TW8, complete genome

gi|40548981|gb|AY502932.1| SARS coronavirus TW9, complete genome

Unresolved cluster 21:

gi|40548909|gb|AY502926.1| SARS coronavirus TW3, complete genome

gi|40548921|gb|AY502927.1| SARS coronavirus TW4, complete genome

Unresolved cluster 22:

gi|38304867|gb|AY282752.2| SARS coronavirus CUHK-Su10, complete genome

Unresolved cluster 23:

gi|33411414|dbj|AP006558.1| SARS coronavirus TWJ genomic RNA, complete genome

Unresolved cluster 24:

gi|38385714|gb|AY461660.1| SARS coronavirus SoD, complete genome

Unresolved cluster 25:

SARS Singapore virus isolate KYK

Unresolved cluster 26:

gi|37624336|gb|AY394993.1| SARS coronavirus HGZ8L2, complete genome

Unresolved cluster 27:

gi|31416305|gb|AY278490.3| SARS coronavirus BJ03, complete genome

Unresolved cluster 28:

gi|37624339|gb|AY394996.1| SARS coronavirus ZS-B, complete genome

gi|37624346|gb|AY395003.1| SARS coronavirus ZS-C, complete genome

Unresolved cluster 29:

gi|37624328|gb|AY394985.1| SARS coronavirus HSZ-Bb, complete genome

gi|37624337|gb|AY394994.1| SARS coronavirus HSZ-Bc, complete genome

gi|37624338|gb|AY394995.1| SARS coronavirus HSZ-Cc, complete genome

Unresolved cluster 30:

gi|40548885|gb|AY502924.1| SARS coronavirus TW11, complete genome

Unresolved cluster 31:

gi|37624330|gb|AY394987.1| SARS coronavirus HZS2-Fb, complete genome

gi|37624334|gb|AY394991.1| SARS coronavirus HZS2-Fc, complete genome

Unresolved cluster 32:

gi|30468042|gb|AY283794.1| SARS coronavirus Sin2500, complete genome

Unresolved cluster 33:

gi|31416290|gb|AY278489.2| SARS coronavirus GD01, complete genome

Unresolved cluster 34:

gi|45644996|gb|AY559082.1| SARS coronavirus Sin852, complete genome

gi|45645000|gb|AY559084.1| SARS coronavirus Sin3765V, complete genome

Unresolved cluster 35:

gi|30421451|gb|AY282752.1| SARS coronavirus CUHK-Su10, complete genome

Unresolved cluster 36:

gi|37624329|gb|AY394986.1| SARS coronavirus HSZ-Cb, complete genome

Unresolved cluster 37:

gi|52546959|gb|AY714217.1| SARS Coronavirus CDC#200301157, complete genome

Unresolved cluster 38:

gi|37624343|gb|AY395000.1| SARS coronavirus LC3, complete genome

Unresolved cluster 39:

gi|30027610|gb|AY278554.2| SARS coronavirus CUHK-W1, complete genome

Unresolved cluster 40:

gi|30275666|gb|AY278488.2| SARS coronavirus BJ01, complete genome

Unresolved cluster 41:

gi|37624322|gb|AY394979.1| SARS coronavirus GZ-C, complete genome

Unresolved cluster 42:

gi|50365700|gb|AY654624.1| SARS coronavirus TJF, complete genome

Unresolved cluster 43:

gi|45644994|gb|AY559081.1| SARS coronavirus Sin842, complete genome

gi|45644998|gb|AY559083.1| SARS coronavirus Sin3408, complete genome

gi|45645024|gb|AY559097.1| SARS coronavirus Sin3408L, complete genome

Unresolved cluster 44:

gi|40795428|gb|AY394850.2| SARS coronavirus WHU, complete genome

Unresolved cluster 45:

gi|37624345|gb|AY395002.1| SARS coronavirus LC5, complete genome

Unresolved cluster 46:

gi|34482137|gb|AY304486.1| SARS coronavirus SZ3, complete genome

Unresolved cluster 47:

gi|32187343|gb|AY323977.1| SARS coronavirus HSR 1, complete genome

Unresolved cluster 48:

gi|41323719|gb|AY390556.1| SARS coronavirus GZ02, complete genome

Unresolved cluster 49:

gi|45645001|gb|AY559085.1| SARS coronavirus Sin848, complete genome

gi|45645022|gb|AY559095.1| SARS coronavirus Sin847, complete genome

Unresolved cluster 50:

gi|40457433|gb|AY463059.1| SARS coronavirus ShanghaiQXC1, complete genome

gi|40457448|gb|AY463060.1| SARS coronavirus ShanghaiQXC2, complete genome

Unresolved cluster 51:

gi|30468043|gb|AY283795.1| SARS coronavirus Sin2677, complete genome

Unresolved cluster 52:

gi|34482146|gb|AY304495.1| SARS coronavirus GZ50, complete genome

Unresolved cluster 53:

gi|38505491|gb|AY485278.1| SARS coronavirus Sino3-11, complete genome

Unresolved cluster 54:

gi|30023963|gb|AY278491.2| SARS coronavirus HKU-39849, complete genome

Unresolved cluster 55:

gi|40795744|gb|AY508724.1| SARS coronavirus NS-1, complete genome

Unresolved cluster 56:

gi|32493130|gb|AY338175.1| SARS coronavirus Taiwan TC2, complete genome

Unresolved cluster 57:

gi|33304219|gb|AY351680.1| SARS coronavirus ZMY 1, complete genome

Unresolved cluster 58:

gi|30468046|gb|AY283798.1| SARS coronavirus Sin2774, complete genome

Unresolved cluster 59:

gi|30910859|gb|AY297028.1| SARS coronavirus ZJ01, complete genome

Unresolved cluster 60:

gi|33188324|gb|AY348314.1| SARS coronavirus Taiwan TC3, complete genome

Unresolved cluster 61:

gi|45645023|gb|AY559096.1| SARS coronavirus Sin850, complete genome

Unresolved cluster 62:

gi|45645021|gb|AY559094.1| SARS coronavirus Sin846, complete genome

Unresolved cluster 63:

gi|45645003|gb|AY559086.1| SARS coronavirus Sin849, complete genome

gi|45645004|gb|AY559087.1| SARS coronavirus Sin3725V, complete genome

Unresolved cluster 64:

gi|32493129|gb|AY338174.1| SARS coronavirus Taiwan TC1, complete genome

Unresolved cluster 65:

gi|29826277|ref|NC_004718.1| SARS coronavirus, complete genome

PCR without restriction digest

Number of unresolved clusters: 26

Unresolved cluster 1:

gi|45645016|gb|AY559091.1| SARS coronavirus SinP4, complete genome

Unresolved cluster 2:

gi|37624339|gb|AY394996.1| SARS coronavirus ZS-B, complete genome

gi|37624346|gb|AY395003.1| SARS coronavirus ZS-C, complete genome

Unresolved cluster 3:

gi|45645003|gb|AY559086.1| SARS coronavirus Sin849, complete genome

Unresolved cluster 4:

gi|45645017|gb|AY559092.1| SARS coronavirus SinP5, complete genome

Unresolved cluster 5:

gi|45645007|gb|AY559088.1| SARS coronavirus SinP1, complete genome

Unresolved cluster 6:

gi|45644994|gb|AY559081.1| SARS coronavirus Sin842, complete genome

Unresolved cluster 7:

gi|37624342|gb|AY394999.1| SARS coronavirus LC2, complete genome

gi|37624343|gb|AY395000.1| SARS coronavirus LC3, complete genome

gi|37624345|gb|AY395002.1| SARS coronavirus LC5, complete genome

Unresolved cluster 8:

gi|31416290|gb|AY278489.2| SARS coronavirus GD01, complete genome

gi|34482137|gb|AY304486.1| SARS coronavirus SZ3, complete genome

gi|34482139|gb|AY304488.1| SARS coronavirus SZ16, complete genome

gi|37624328|gb|AY394985.1| SARS coronavirus HSZ-Bb, complete genome

gi|37624329|gb|AY394986.1| SARS coronavirus HSZ-Cb, complete genome

gi|37624337|gb|AY394994.1| SARS coronavirus HSZ-Bc, complete genome

gi|37624338|gb|AY394995.1| SARS coronavirus HSZ-Cc, complete genome

gi|41323719|gb|AY390556.1| SARS coronavirus GZ02, complete genome

Unresolved cluster 9:

gi|37624321|gb|AY394978.1| SARS coronavirus GZ-B, complete genome

Unresolved cluster 10:

gi|45644996|gb|AY559082.1| SARS coronavirus Sin852, complete genome

Unresolved cluster 11:

gi|37960831|gb|AY313906.1| SARS coronavirus GD69, complete genome

Unresolved cluster 12:

gi|31873092|gb|AY321118.1| SARS coronavirus TWC, complete genome

gi|35396382|gb|AY394850.1| SARS coronavirus WHU, complete genome

gi|38231932|gb|AY357075.1| SARS coronavirus PUMC02, complete genome

gi|38505491|gb|AY485278.1| SARS coronavirus Sino3-11, complete genome

gi|40795428|gb|AY394850.2| SARS coronavirus WHU, complete genome

Unresolved cluster 13:

gi|37624322|gb|AY394979.1| SARS coronavirus GZ-C, complete genome

Unresolved cluster 14:

gi|40457448|gb|AY463060.1| SARS coronavirus ShanghaiQXC2, complete genome

Unresolved cluster 15:

gi|49176846|gb|AY595412.1| SARS coronavirus LLJ-2004, complete genome

Unresolved cluster 16:

gi|33411414|dbj|AP006558.1| SARS coronavirus TWJ genomic RNA, complete genome

gi|40548885|gb|AY502924.1| SARS coronavirus TW11, complete genome

Unresolved cluster 17:

gi|30468043|gb|AY283795.1| SARS coronavirus Sin2677, complete genome

Unresolved cluster 18:

gi|30468045|gb|AY283797.1| SARS coronavirus Sin2748, complete genome

Unresolved cluster 19:

gi|33304219|gb|AY351680.1| SARS coronavirus ZMY 1, complete genome

Unresolved cluster 20:

gi|30910859|gb|AY297028.1| SARS coronavirus ZJ01, complete genome

Unresolved cluster 21:

SARS Singapore virus isolate KYK

Unresolved cluster 22:

gi|45645013|gb|AY559090.1| SARS coronavirus SinP3, complete genome

Unresolved cluster 23:

gi|45645024|gb|AY559097.1| SARS coronavirus Sin3408L, complete genome

Unresolved cluster 24:

gi|29826277|ref|NC_004718.1| SARS coronavirus, complete genome

gi|30023963|gb|AY278491.2| SARS coronavirus HKU-39849, complete genome

gi|30027610|gb|AY278554.2| SARS coronavirus CUHK-W1, complete genome

gi|30027617|gb|AY278741.1| SARS coronavirus Urbani, complete genome

gi|30271926|ref|NC_004718.3| SARS coronavirus, complete genome

gi|30275666|gb|AY278488.2| SARS coronavirus BJ01, complete genome

gi|30421451|gb|AY282752.1| SARS coronavirus CUHK-Su10, complete genome

gi|30468042|gb|AY283794.1| SARS coronavirus Sin2500, complete genome

gi|30468044|gb|AY283796.1| SARS coronavirus Sin2679, complete genome

gi|30468046|gb|AY283798.1| SARS coronavirus Sin2774, complete genome

gi|30698326|gb|AY291451.1| SARS coronavirus TW1, complete genome

gi|31416292|gb|AY278487.3| SARS coronavirus BJ02, complete genome

gi|31416305|gb|AY278490.3| SARS coronavirus BJ03, complete genome

gi|31581502|gb|AY291315.1| SARS coronavirus Frankfurt 1, complete genome

gi|32187343|gb|AY323977.1| SARS coronavirus HSR 1, complete genome

gi|32493129|gb|AY338174.1| SARS coronavirus Taiwan TC1, complete genome

gi|32493130|gb|AY338175.1| SARS coronavirus Taiwan TC2, complete genome

gi|33114190|gb|AY345986.1| SARS coronavirus CUHK-AG01, complete genome

gi|33114214|gb|AY345988.1| SARS coronavirus CUHK-AG03, complete genome

gi|33115118|gb|AY323977.2| SARS coronavirus HSR 1, complete genome

gi|33188324|gb|AY348314.1| SARS coronavirus Taiwan TC3, complete genome

gi|33411399|dbj|AP006557.1| SARS coronavirus TWH genomic RNA, complete genome

gi|33411429|dbj|AP006559.1| SARS coronavirus TWK genomic RNA, complete genome

gi|33411444|dbj|AP006560.1| SARS coronavirus TWS genomic RNA, complete genome

gi|33411459|dbj|AP006561.1| SARS coronavirus TWY genomic RNA, complete genome

gi|33518725|gb|AY362699.1| SARS coronavirus TWC3, complete genome

gi|33578015|gb|AY310120.1| SARS coronavirus FRA, complete genome

gi|34482146|gb|AY304495.1| SARS coronavirus GZ50, complete genome

gi|37361915|gb|AY283798.2| SARS coronavirus Sin2774, complete genome

gi|37576845|gb|AY427439.1| SARS coronavirus AS, complete genome

gi|37624326|gb|AY394983.1| SARS coronavirus HSZ2-A, complete genome

gi|37624330|gb|AY394987.1| SARS coronavirus HZS2-Fb, complete genome

gi|37624332|gb|AY394989.1| SARS coronavirus HZS2-D, complete genome

gi|37624333|gb|AY394990.1| SARS coronavirus HZS2-E, complete genome

gi|37624334|gb|AY394991.1| SARS coronavirus HZS2-Fc, complete genome

gi|37624335|gb|AY394992.1| SARS coronavirus HZS2-C, complete genome

gi|37624336|gb|AY394993.1| SARS coronavirus HGZ8L2, complete genome

gi|37624341|gb|AY394998.1| SARS coronavirus LC1, complete genome

gi|38231927|gb|AY350750.1| SARS coronavirus PUMC01, complete genome

gi|38231937|gb|AY357076.1| SARS coronavirus PUMC03, complete genome

gi|38304867|gb|AY282752.2| SARS coronavirus CUHK-Su10, complete genome

gi|38385714|gb|AY461660.1| SARS coronavirus SoD, complete genome

gi|38505482|gb|AY485277.1| SARS coronavirus Sino1-11, complete genome

gi|40457433|gb|AY463059.1| SARS coronavirus ShanghaiQXC1, complete genome

gi|40548873|gb|AY502923.1| SARS coronavirus TW10, complete genome

gi|40548909|gb|AY502926.1| SARS coronavirus TW3, complete genome

gi|40548921|gb|AY502927.1| SARS coronavirus TW4, complete genome

gi|40548933|gb|AY502928.1| SARS coronavirus TW5, complete genome

gi|40548945|gb|AY502929.1| SARS coronavirus TW6, complete genome

gi|40548957|gb|AY502930.1| SARS coronavirus TW7, complete genome

gi|40548969|gb|AY502931.1| SARS coronavirus TW8, complete genome

gi|40548981|gb|AY502932.1| SARS coronavirus TW9, complete genome

gi|40795744|gb|AY508724.1| SARS coronavirus NS-1, complete genome

gi|45644998|gb|AY559083.1| SARS coronavirus Sin3408, complete genome

gi|45645000|gb|AY559084.1| SARS coronavirus Sin3765V, complete genome

gi|45645001|gb|AY559085.1| SARS coronavirus Sin848, complete genome

gi|45645004|gb|AY559087.1| SARS coronavirus Sin3725V, complete genome

gi|45645019|gb|AY559093.1| SARS coronavirus Sin845, complete genome

gi|45645022|gb|AY559095.1| SARS coronavirus Sin847, complete genome

gi|45645023|gb|AY559096.1| SARS coronavirus Sin850, complete genome

gi|50365700|gb|AY654624.1| SARS coronavirus TJF, complete genome

gi|52546959|gb|AY714217.1| SARS Coronavirus CDC#200301157, complete genome

Unresolved cluster 25:

gi|45645021|gb|AY559094.1| SARS coronavirus Sin846, complete genome

Unresolved cluster 26:

gi|45645010|gb|AY559089.1| SARS coronavirus SinP2, complete genome

PCR-RFLP

Number of unresolved clusters: 75

Unresolved cluster 1:

gi|33411459|dbj|AP006561.1| SARS coronavirus TWY genomic RNA, complete genome

gi|40548873|gb|AY502923.1| SARS coronavirus TW10, complete genome

Unresolved cluster 2:

gi|37624342|gb|AY394999.1| SARS coronavirus LC2, complete genome

Unresolved cluster 3:

gi|45645019|gb|AY559093.1| SARS coronavirus Sin845, complete genome

Unresolved cluster 4:

gi|45645000|gb|AY559084.1| SARS coronavirus Sin3765V, complete genome

Unresolved cluster 5:

gi|31416292|gb|AY278487.3| SARS coronavirus BJ02, complete genome

Unresolved cluster 6:

gi|34482139|gb|AY304488.1| SARS coronavirus SZ16, complete genome

Unresolved cluster 7:

gi|37624321|gb|AY394978.1| SARS coronavirus GZ-B, complete genome

Unresolved cluster 8:

gi|37960831|gb|AY313906.1| SARS coronavirus GD69, complete genome

Unresolved cluster 9:

gi|30698326|gb|AY291451.1| SARS coronavirus TW1, complete genome

Unresolved cluster 10:

gi|40457433|gb|AY463059.1| SARS coronavirus ShanghaiQXC1, complete genome

Unresolved cluster 11:

gi|37624343|gb|AY395000.1| SARS coronavirus LC3, complete genome

gi|37624345|gb|AY395002.1| SARS coronavirus LC5, complete genome

Unresolved cluster 12:

gi|37624333|gb|AY394990.1| SARS coronavirus HZS2-E, complete genome

Unresolved cluster 13:

gi|49176846|gb|AY595412.1| SARS coronavirus LLJ-2004, complete genome

Unresolved cluster 14:

gi|33411414|dbj|AP006558.1| SARS coronavirus TWJ genomic RNA, complete genome

Unresolved cluster 15:

SARS Singapore virus isolate KYK

Unresolved cluster 16:

gi|45645013|gb|AY559090.1| SARS coronavirus SinP3, complete genome

Unresolved cluster 17:

gi|31873092|gb|AY321118.1| SARS coronavirus TWC, complete genome

gi|40795428|gb|AY394850.2| SARS coronavirus WHU, complete genome

Unresolved cluster 18:

gi|45645024|gb|AY559097.1| SARS coronavirus Sin3408L, complete genome

Unresolved cluster 19:

gi|31416305|gb|AY278490.3| SARS coronavirus BJ03, complete genome

Unresolved cluster 20:

gi|37624328|gb|AY394985.1| SARS coronavirus HSZ-Bb, complete genome

gi|37624337|gb|AY394994.1| SARS coronavirus HSZ-Bc, complete genome

Unresolved cluster 21:

gi|45645017|gb|AY559092.1| SARS coronavirus SinP5, complete genome

Unresolved cluster 22:

gi|40548885|gb|AY502924.1| SARS coronavirus TW11, complete genome

Unresolved cluster 23:

gi|30468042|gb|AY283794.1| SARS coronavirus Sin2500, complete genome

Unresolved cluster 24:

gi|31416290|gb|AY278489.2| SARS coronavirus GD01, complete genome

Unresolved cluster 25:

gi|45644996|gb|AY559082.1| SARS coronavirus Sin852, complete genome

Unresolved cluster 26:

gi|30275666|gb|AY278488.2| SARS coronavirus BJ01, complete genome

Unresolved cluster 27:

gi|37624322|gb|AY394979.1| SARS coronavirus GZ-C, complete genome

Unresolved cluster 28:

gi|30421451|gb|AY282752.1| SARS coronavirus CUHK-Su10, complete genome

gi|30468044|gb|AY283796.1| SARS coronavirus Sin2679, complete genome

gi|32187343|gb|AY323977.1| SARS coronavirus HSR 1, complete genome

gi|33114190|gb|AY345986.1| SARS coronavirus CUHK-AG01, complete genome

gi|33115118|gb|AY323977.2| SARS coronavirus HSR 1, complete genome

gi|33411399|dbj|AP006557.1| SARS coronavirus TWH genomic RNA, complete genome

gi|33411429|dbj|AP006559.1| SARS coronavirus TWK genomic RNA, complete genome

gi|33411444|dbj|AP006560.1| SARS coronavirus TWS genomic RNA, complete genome

gi|33518725|gb|AY362699.1| SARS coronavirus TWC3, complete genome

gi|37576845|gb|AY427439.1| SARS coronavirus AS, complete genome

gi|37624341|gb|AY394998.1| SARS coronavirus LC1, complete genome

gi|38231927|gb|AY350750.1| SARS coronavirus PUMC01, complete genome

gi|38304867|gb|AY282752.2| SARS coronavirus CUHK-Su10, complete genome

gi|40548933|gb|AY502928.1| SARS coronavirus TW5, complete genome

gi|40548945|gb|AY502929.1| SARS coronavirus TW6, complete genome

gi|40548957|gb|AY502930.1| SARS coronavirus TW7, complete genome

gi|40548969|gb|AY502931.1| SARS coronavirus TW8, complete genome

gi|40548981|gb|AY502932.1| SARS coronavirus TW9, complete genome

gi|52546959|gb|AY714217.1| SARS Coronavirus CDC#200301157, complete genome

Unresolved cluster 29:

gi|40548921|gb|AY502927.1| SARS coronavirus TW4, complete genome

Unresolved cluster 30:

gi|34482137|gb|AY304486.1| SARS coronavirus SZ3, complete genome

Unresolved cluster 31:

gi|38505491|gb|AY485278.1| SARS coronavirus Sino3-11, complete genome

Unresolved cluster 32:

gi|30023963|gb|AY278491.2| SARS coronavirus HKU-39849, complete genome

Unresolved cluster 33:

gi|40795744|gb|AY508724.1| SARS coronavirus NS-1, complete genome

Unresolved cluster 34:

gi|30468045|gb|AY283797.1| SARS coronavirus Sin2748, complete genome

Unresolved cluster 35:

gi|30468046|gb|AY283798.1| SARS coronavirus Sin2774, complete genome

Unresolved cluster 36:

gi|30910859|gb|AY297028.1| SARS coronavirus ZJ01, complete genome

Unresolved cluster 37:

gi|45645023|gb|AY559096.1| SARS coronavirus Sin850, complete genome

Unresolved cluster 38:

gi|45645021|gb|AY559094.1| SARS coronavirus Sin846, complete genome

Unresolved cluster 39:

gi|32493129|gb|AY338174.1| SARS coronavirus Taiwan TC1, complete genome

Unresolved cluster 40:

gi|45644998|gb|AY559083.1| SARS coronavirus Sin3408, complete genome

Unresolved cluster 41:

gi|45645010|gb|AY559089.1| SARS coronavirus SinP2, complete genome

Unresolved cluster 42:

gi|45645016|gb|AY559091.1| SARS coronavirus SinP4, complete genome

Unresolved cluster 43:

gi|45645003|gb|AY559086.1| SARS coronavirus Sin849, complete genome

Unresolved cluster 44:

gi|45645004|gb|AY559087.1| SARS coronavirus Sin3725V, complete genome

Unresolved cluster 45:

gi|37624338|gb|AY394995.1| SARS coronavirus HSZ-Cc, complete genome

Unresolved cluster 46:

gi|30027617|gb|AY278741.1| SARS coronavirus Urbani, complete genome

Unresolved cluster 47:

gi|35396382|gb|AY394850.1| SARS coronavirus WHU, complete genome

Unresolved cluster 48:

gi|37624335|gb|AY394992.1| SARS coronavirus HZS2-C, complete genome

Unresolved cluster 49:

gi|31581502|gb|AY291315.1| SARS coronavirus Frankfurt 1, complete genome

gi|33578015|gb|AY310120.1| SARS coronavirus FRA, complete genome

Unresolved cluster 50:

gi|38231937|gb|AY357076.1| SARS coronavirus PUMC03, complete genome

gi|38505482|gb|AY485277.1| SARS coronavirus Sino1-11, complete genome

Unresolved cluster 51:

gi|38385714|gb|AY461660.1| SARS coronavirus SoD, complete genome

Unresolved cluster 52:

gi|37624336|gb|AY394993.1| SARS coronavirus HGZ8L2, complete genome

Unresolved cluster 53:

gi|37624339|gb|AY394996.1| SARS coronavirus ZS-B, complete genome

gi|37624346|gb|AY395003.1| SARS coronavirus ZS-C, complete genome

Unresolved cluster 54:

gi|45645007|gb|AY559088.1| SARS coronavirus SinP1, complete genome

Unresolved cluster 55:

gi|37624330|gb|AY394987.1| SARS coronavirus HZS2-Fb, complete genome

gi|37624334|gb|AY394991.1| SARS coronavirus HZS2-Fc, complete genome

Unresolved cluster 56:

gi|38231932|gb|AY357075.1| SARS coronavirus PUMC02, complete genome

Unresolved cluster 57:

gi|45644994|gb|AY559081.1| SARS coronavirus Sin842, complete genome

Unresolved cluster 58:

gi|37624329|gb|AY394986.1| SARS coronavirus HSZ-Cb, complete genome

Unresolved cluster 59:

gi|30027610|gb|AY278554.2| SARS coronavirus CUHK-W1, complete genome

Unresolved cluster 60:

gi|40457448|gb|AY463060.1| SARS coronavirus ShanghaiQXC2, complete genome

Unresolved cluster 61:

gi|50365700|gb|AY654624.1| SARS coronavirus TJF, complete genome

Unresolved cluster 62:

gi|40795428|gb|AY394850.2| SARS coronavirus WHU, complete genome

Unresolved cluster 63:

gi|33114214|gb|AY345988.1| SARS coronavirus CUHK-AG03, complete genome

Unresolved cluster 64:

gi|40548909|gb|AY502926.1| SARS coronavirus TW3, complete genome

Unresolved cluster 65:

gi|41323719|gb|AY390556.1| SARS coronavirus GZ02, complete genome

Unresolved cluster 66:

gi|30271926|ref|NC_004718.3| SARS coronavirus, complete genome

Unresolved cluster 67:

gi|30468043|gb|AY283795.1| SARS coronavirus Sin2677, complete genome

Unresolved cluster 68:

gi|45645001|gb|AY559085.1| SARS coronavirus Sin848, complete genome

gi|45645022|gb|AY559095.1| SARS coronavirus Sin847, complete genome

Unresolved cluster 69:

gi|34482146|gb|AY304495.1| SARS coronavirus GZ50, complete genome

Unresolved cluster 70:

gi|33304219|gb|AY351680.1| SARS coronavirus ZMY 1, complete genome

Unresolved cluster 71:

gi|32493130|gb|AY338175.1| SARS coronavirus Taiwan TC2, complete genome

Unresolved cluster 72:

gi|33188324|gb|AY348314.1| SARS coronavirus Taiwan TC3, complete genome

Unresolved cluster 73:

gi|37361915|gb|AY283798.2| SARS coronavirus Sin2774, complete genome

Unresolved cluster 74:

gi|37624326|gb|AY394983.1| SARS coronavirus HSZ2-A, complete genome

gi|37624332|gb|AY394989.1| SARS coronavirus HZS2-D, complete genome

Unresolved cluster 75:

gi|29826277|ref|NC_004718.1| SARS coronavirus, complete genome

The following are the unresolved clusters for mumps virus for SNPs and PCR-RFLPs.

Mumps SNP analysis with min_len_upstream= min_len_downstream=7:

Number of unresolved clusters: 11

Unresolved cluster 1:

gi|19070168|gb|AF345290.1| Mumps virus (STRAIN JERYL-LYNN) live vaccine minor component JL2, complete genome

Unresolved cluster 2:

gi|15077508|gb|AF338106.1| Mumps virus (STRAIN JERYL-LYNN) live vaccine major component, complete genome

gi|7861760|gb|AF201473.1|AF201473 Mumps virus, complete genome

Unresolved cluster 3:

gi|9695415|ref|NC_002200.1| Mumps virus, complete genome

Unresolved cluster 4:

gi|50812713|gb|AY681495.1| Mumps virus strain PetroNov genotype H, complete genome

Unresolved cluster 5:

gi|50404164|gb|AY669145.1| Mumps virus genotype C, complete genome

Unresolved cluster 6:

gi|46254660|gb|AY508995.1| Mumps virus strain L3/Russia/Vector, complete genome

Unresolved cluster 7:

gi|32172464|gb|AY309060.1| Mumps virus isolate Dg1062/Korea/98, complete genome

Unresolved cluster 8:

gi|14325886|gb|AF314558.1| Mumps virus strain SIPAR 02, complete genome

gi|14325886|gb|AF314558.1|AF314558 Mumps virus, complete genome

gi|14325896|gb|AF314559.1|AF314559 Mumps virus, complete genome

gi|14325906|gb|AF314560.1|AF314560 Mumps virus isolate 87 1004, complete genome

gi|14325916|gb|AF314561.1|AF314561 Mumps virus strain Biken, complete genome

gi|14325926|gb|AF314562.1|AF314562 Mumps virus isolate 87 1005, complete genome

Unresolved cluster 9:

gi|11545407|gb|AF280799.1|AF280799 Mumps virus strain Glouc1/UK96, complete genome

Unresolved cluster 10:

gi|34224045|gb|AF467767.2| Mumps virus isolate 88-1961, complete genome

Unresolved cluster 11:

gi|18643326|gb|AF467767.1| Mumps virus isolate 88-1961, complete genome

For mumps virus, PCR-RFLP with restriction digest with num_restriction_enzymes=1 using the 99 possible enzymes listed at http://est.llnl.gov/forensics yields the following unresolved clusters:

Unresolved cluster 1:

gi|19070168|gb|AF345290.1| Mumps virus (STRAIN JERYL-LYNN) live vaccine minor component JL2, complete genome

Unresolved cluster 2:

gi|14325926|gb|AF314562.1|AF314562 Mumps virus isolate 87 1005, complete genome

Unresolved cluster 3:

gi|9695415|ref|NC_002200.1| Mumps virus, complete genome

Unresolved cluster 4:

gi|50404164|gb|AY669145.1| Mumps virus genotype C, complete genome

Unresolved cluster 5:

gi|32172464|gb|AY309060.1| Mumps virus isolate Dg1062/Korea/98, complete genome

Unresolved cluster 6:

gi|14325886|gb|AF314558.1| Mumps virus strain SIPAR 02, complete genome

gi|14325886|gb|AF314558.1|AF314558 Mumps virus, complete genome

gi|14325906|gb|AF314560.1|AF314560 Mumps virus isolate 87 1004, complete genome

gi|14325916|gb|AF314561.1|AF314561 Mumps virus strain Biken, complete genome

Unresolved cluster 7:

gi|34224045|gb|AF467767.2| Mumps virus isolate 88-1961, complete genome

Unresolved cluster 8:

gi|15077508|gb|AF338106.1| Mumps virus (STRAIN JERYL-LYNN) live vaccine major component, complete genome

gi|7861760|gb|AF201473.1|AF201473 Mumps virus, complete genome

Unresolved cluster 9:

gi|50812713|gb|AY681495.1| Mumps virus strain PetroNov genotype H, complete genome

Unresolved cluster 10:

gi|46254660|gb|AY508995.1| Mumps virus strain L3/Russia/Vector, complete genome

Unresolved cluster 11:

gi|11545407|gb|AF280799.1|AF280799 Mumps virus strain Glouc1/UK96, complete genome

Unresolved cluster 12:

gi|14325896|gb|AF314559.1|AF314559 Mumps virus, complete genome

Unresolved cluster 13:

gi|18643326|gb|AF467767.1| Mumps virus isolate 88-1961, complete genome
