# Supplementary material for: Software for optimization of SNP and PCR-RFLP genotyping to discriminate many genomes with the fewest assays
Source: BMC Genomics. 2005 May 16;6:73. doi: 10.1186/1471-2164-6-73 (PMC1156889; doi:10.1186/1471-2164-6-73)
Supplement: Additional File 1 — List of genomes used in the analyses. Description of additional data files provided at Digest*organism_name NoDigest*organism_name SNP*organism_name The * refers to the files indicated in Table 2. Digest* is for PCR-RFLP with num_restriction_enzymes = 1, and NoDigest* is for PCR-RFLP with num_restriction_enzymes = 0. The NoDigest* results are not given for mumps, since there was not adequate variation using this method for forensic discrimination of these input sequences, as indicated in Table 4. The multiple sequence alignment files used in these analyses for SARS and mumps viruses are also available for download. There are a total of 27 files containing all the microbial forensic results and data described above. All are in text format, and can be found at . [file 1471-2164-6-73-S1.doc]

Additional File 1: List of genomes used in the analyses

Genomes included in forensic analyses:

102 genomes downloaded from Genbank on December 28, 2004

gi|30271926|ref|NC_004718.3| SARS coronavirus, complete genome

SARS Singapore virus isolate KYK

gi|29826277|ref|NC_004718.1| SARS coronavirus, complete genome

gi|33411399|dbj|AP006557.1| SARS coronavirus TWH genomic RNA, complete genome

gi|33411414|dbj|AP006558.1| SARS coronavirus TWJ genomic RNA, complete genome

gi|33411429|dbj|AP006559.1| SARS coronavirus TWK genomic RNA, complete genome

gi|33411444|dbj|AP006560.1| SARS coronavirus TWS genomic RNA, complete genome

gi|33411459|dbj|AP006561.1| SARS coronavirus TWY genomic RNA, complete genome

gi|33518725|gb|AY362699.1| SARS coronavirus TWC3, complete genome

gi|35396382|gb|AY394850.1| SARS coronavirus WHU, complete genome

gi|40795428|gb|AY394850.2| SARS coronavirus WHU, complete genome

gi|37576845|gb|AY427439.1| SARS coronavirus AS, complete genome

gi|33578015|gb|AY310120.1| SARS coronavirus FRA, complete genome

gi|30910859|gb|AY297028.1| SARS coronavirus ZJ01, complete genome

gi|32493129|gb|AY338174.1| SARS coronavirus Taiwan TC1, complete genome

gi|32493130|gb|AY338175.1| SARS coronavirus Taiwan TC2, complete genome

gi|30027617|gb|AY278741.1| SARS coronavirus Urbani, complete genome

gi|30023963|gb|AY278491.2| SARS coronavirus HKU-39849, complete genome

gi|31416292|gb|AY278487.3| SARS coronavirus BJ02, complete genome

gi|30275666|gb|AY278488.2| SARS coronavirus BJ01, complete genome

gi|31416305|gb|AY278490.3| SARS coronavirus BJ03, complete genome

gi|30027610|gb|AY278554.2| SARS coronavirus CUHK-W1, complete genome

gi|34482137|gb|AY304486.1| SARS coronavirus SZ3, complete genome

gi|34482139|gb|AY304488.1| SARS coronavirus SZ16, complete genome

gi|34482146|gb|AY304495.1| SARS coronavirus GZ50, complete genome

gi|31581502|gb|AY291315.1| SARS coronavirus Frankfurt 1, complete genome

gi|33188324|gb|AY348314.1| SARS coronavirus Taiwan TC3, complete genome

gi|31873092|gb|AY321118.1| SARS coronavirus TWC, complete genome

gi|32187343|gb|AY323977.1| SARS coronavirus HSR 1, complete genome

gi|33115118|gb|AY323977.2| SARS coronavirus HSR 1, complete genome

gi|30468046|gb|AY283798.1| SARS coronavirus Sin2774, complete genome

gi|37361915|gb|AY283798.2| SARS coronavirus Sin2774, complete genome

gi|30468045|gb|AY283797.1| SARS coronavirus Sin2748, complete genome

gi|30468044|gb|AY283796.1| SARS coronavirus Sin2679, complete genome

gi|30468043|gb|AY283795.1| SARS coronavirus Sin2677, complete genome

gi|30468042|gb|AY283794.1| SARS coronavirus Sin2500, complete genome

gi|31416290|gb|AY278489.2| SARS coronavirus GD01, complete genome

gi|37960831|gb|AY313906.1| SARS coronavirus GD69, complete genome

gi|30421451|gb|AY282752.1| SARS coronavirus CUHK-Su10, complete genome

gi|38304867|gb|AY282752.2| SARS coronavirus CUHK-Su10, complete genome

gi|30698326|gb|AY291451.1| SARS coronavirus TW1, complete genome

gi|33304219|gb|AY351680.1| SARS coronavirus ZMY 1, complete genome

gi|37624321|gb|AY394978.1| SARS coronavirus GZ-B, complete genome

gi|37624322|gb|AY394979.1| SARS coronavirus GZ-C, complete genome

gi|37624326|gb|AY394983.1| SARS coronavirus HSZ2-A, complete genome

gi|37624328|gb|AY394985.1| SARS coronavirus HSZ-Bb, complete genome

gi|37624329|gb|AY394986.1| SARS coronavirus HSZ-Cb, complete genome

gi|37624330|gb|AY394987.1| SARS coronavirus HZS2-Fb, complete genome

gi|37624332|gb|AY394989.1| SARS coronavirus HZS2-D, complete genome

gi|37624333|gb|AY394990.1| SARS coronavirus HZS2-E, complete genome

gi|37624334|gb|AY394991.1| SARS coronavirus HZS2-Fc, complete genome

gi|37624335|gb|AY394992.1| SARS coronavirus HZS2-C, complete genome

gi|37624336|gb|AY394993.1| SARS coronavirus HGZ8L2, complete genome

gi|37624337|gb|AY394994.1| SARS coronavirus HSZ-Bc, complete genome

gi|37624338|gb|AY394995.1| SARS coronavirus HSZ-Cc, complete genome

gi|37624339|gb|AY394996.1| SARS coronavirus ZS-B, complete genome

gi|37624341|gb|AY394998.1| SARS coronavirus LC1, complete genome

gi|37624342|gb|AY394999.1| SARS coronavirus LC2, complete genome

gi|37624343|gb|AY395000.1| SARS coronavirus LC3, complete genome

gi|37624345|gb|AY395002.1| SARS coronavirus LC5, complete genome

gi|37624346|gb|AY395003.1| SARS coronavirus ZS-C, complete genome

gi|41323719|gb|AY390556.1| SARS coronavirus GZ02, complete genome

gi|40795744|gb|AY508724.1| SARS coronavirus NS-1, complete genome

gi|33114190|gb|AY345986.1| SARS coronavirus CUHK-AG01, complete genome

gi|33114214|gb|AY345988.1| SARS coronavirus CUHK-AG03, complete genome

gi|38231927|gb|AY350750.1| SARS coronavirus PUMC01, complete genome

gi|38231932|gb|AY357075.1| SARS coronavirus PUMC02, complete genome

gi|38231937|gb|AY357076.1| SARS coronavirus PUMC03, complete genome

gi|38385714|gb|AY461660.1| SARS coronavirus SoD, complete genome

gi|38505491|gb|AY485278.1| SARS coronavirus Sino3-11, complete genome

gi|38505482|gb|AY485277.1| SARS coronavirus Sino1-11, complete genome

gi|40457433|gb|AY463059.1| SARS coronavirus ShanghaiQXC1, complete genome

gi|40457448|gb|AY463060.1| SARS coronavirus ShanghaiQXC2, complete genome

gi|40548873|gb|AY502923.1| SARS coronavirus TW10, complete genome

gi|40548885|gb|AY502924.1| SARS coronavirus TW11, complete genome

gi|40548909|gb|AY502926.1| SARS coronavirus TW3, complete genome

gi|40548921|gb|AY502927.1| SARS coronavirus TW4, complete genome

gi|40548933|gb|AY502928.1| SARS coronavirus TW5, complete genome

gi|40548945|gb|AY502929.1| SARS coronavirus TW6, complete genome

gi|40548957|gb|AY502930.1| SARS coronavirus TW7, complete genome

gi|40548969|gb|AY502931.1| SARS coronavirus TW8, complete genome

gi|40548981|gb|AY502932.1| SARS coronavirus TW9, complete genome

gi|50365700|gb|AY654624.1| SARS coronavirus TJF, complete genome

gi|45644994|gb|AY559081.1| SARS coronavirus Sin842, complete genome

gi|45644996|gb|AY559082.1| SARS coronavirus Sin852, complete genome

gi|45644998|gb|AY559083.1| SARS coronavirus Sin3408, complete genome

gi|45645000|gb|AY559084.1| SARS coronavirus Sin3765V, complete genome

gi|45645001|gb|AY559085.1| SARS coronavirus Sin848, complete genome

gi|45645003|gb|AY559086.1| SARS coronavirus Sin849, complete genome

gi|45645004|gb|AY559087.1| SARS coronavirus Sin3725V, complete genome

gi|45645007|gb|AY559088.1| SARS coronavirus SinP1, complete genome

gi|45645010|gb|AY559089.1| SARS coronavirus SinP2, complete genome

gi|45645013|gb|AY559090.1| SARS coronavirus SinP3, complete genome

gi|45645016|gb|AY559091.1| SARS coronavirus SinP4, complete genome

gi|45645017|gb|AY559092.1| SARS coronavirus SinP5, complete genome

gi|45645019|gb|AY559093.1| SARS coronavirus Sin845, complete genome

gi|45645021|gb|AY559094.1| SARS coronavirus Sin846, complete genome

gi|45645022|gb|AY559095.1| SARS coronavirus Sin847, complete genome

gi|45645023|gb|AY559096.1| SARS coronavirus Sin850, complete genome

gi|45645024|gb|AY559097.1| SARS coronavirus Sin3408L, complete genome

gi|49176846|gb|AY595412.1| SARS coronavirus LLJ-2004, complete genome

gi|52546959|gb|AY714217.1| SARS Coronavirus CDC#200301157, complete genome

17 genomes downloaded from Genbank on December 28, 2004:

gi|14325926|gb|AF314562.1|AF314562 Mumps virus isolate 87 1005, complete genome

gi|14325916|gb|AF314561.1|AF314561 Mumps virus strain Biken, complete genome

gi|46254660|gb|AY508995.1| Mumps virus strain L3/Russia/Vector, complete genome

gi|14325886|gb|AF314558.1| Mumps virus strain SIPAR 02, complete genome

gi|50812713|gb|AY681495.1| Mumps virus strain PetroNov genotype H, complete genome

gi|50404164|gb|AY669145.1| Mumps virus genotype C, complete genome

gi|9695415|ref|NC_002200.1| Mumps virus, complete genome

gi|19070168|gb|AF345290.1| Mumps virus (STRAIN JERYL-LYNN) live vaccine minor component JL2, complete genome

gi|15077508|gb|AF338106.1| Mumps virus (STRAIN JERYL-LYNN) live vaccine major component, complete genome

gi|14325906|gb|AF314560.1|AF314560 Mumps virus isolate 87 1004, complete genome

gi|14325896|gb|AF314559.1|AF314559 Mumps virus, complete genome

gi|14325886|gb|AF314558.1|AF314558 Mumps virus, complete genome

gi|11545407|gb|AF280799.1|AF280799 Mumps virus strain Glouc1/UK96, complete genome

gi|7861760|gb|AF201473.1|AF201473 Mumps virus, complete genome

gi|18643326|gb|AF467767.1| Mumps virus isolate 88-1961, complete genome

gi|34224045|gb|AF467767.2| Mumps virus isolate 88-1961, complete genome

gi|32172464|gb|AY309060.1| Mumps virus isolate Dg1062/Korea/98, complete genome
